# Poor Vaccine Effectiveness against Influenza B-Related Severe Acute Respiratory Infection in a Temperate North Indian State (2019–2020): A Call for Further Data for Possible Vaccines with Closer Match

**DOI:** 10.3390/vaccines9101094

**Published:** 2021-09-28

**Authors:** Hyder Mir, Inaamul Haq, Parvaiz A. Koul

**Affiliations:** 1 Influenza Lab, Internal and Pulmonary Medicine, SKIMS, Soura, Srinagar 190011, J&K, India; mirhyder8@gmail.com; 2 Department of Community Medicine, Government Medical College, Srinagar 190010, J&K, India; haqinaam@yahoo.co.in; 3 Department of Internal and Pulmonary Medicine, Sheri Kashmir Institute of Medical Sciences, Soura, Srinagar 190011, J&K, India

**Keywords:** influenza, vaccine, vaccine effectiveness, influenza B, SARI

## Abstract

**Background:** Influenza vaccine uptake in India is poor, and scant data exist regarding the effectiveness of influenza vaccine against hospitalization. **Methods:** From October 2019 to March 2020, vaccination status of 1219 patients (males *n* = 571, aged 5–107 years; median, 50 years) hospitalized with severe acute respiratory illness (SARI) was assessed. The patients were tested for influenza viruses and their subtypes by RT PCR. Sequencing of the HA gene was performed. Vaccine effectiveness (VE) against influenza subtypes was estimated by the test negative design. **Results:** A total of 336 (27.5%) patients were influenza-positive, with influenza B/Victoria accounting for 49.7% (*n* = 167), followed by influenza A/H1N1 (47.6%; *n* = 155) and influenza A/H3N2 (4.4%; *n* = 15). About 6.8% and 8.6% of the influenza-positive and influenza-negative patients, respectively, had been vaccinated. Adjusted VE for any influenza strain was 13% (95% CI −42 to 47), which for influenza B was 0%. HA sequencing revealed that influenza B samples mainly belonged to subclade V1A.3/133R with deletion of residues 163–165, as against the 2-aa deletion in influenza B/Colorado/06/2017 strain, contained in the vaccine. VE for influenza A/H1N1 was 55%. **Conclusions:** Poor VE due to a genetic mismatch between the circulating strain and the vaccine strain calls for efforts to reduce the mismatch.

## 1. Introduction

Influenza viral infection remains an important cause of morbidity and mortality, and in 2018, it was estimated to result in up to 409,111 deaths (95% credibility interval 291,243–645,832) [1]. In a recent meta-analysis, 32,126,000 (95% CI 20,484,000–46,129,000) influenza-associated lower respiratory infection episodes and 5,678,000 (95% CI 3,205,000–9,432,000) LRI hospitalizations were estimated to occur each year among adults [2]. While intermittent outbreaks of influenza in India have been reported in the past century, the virus has been recognized as an important and common pathogen rather recently, with increasing reports of influenza virus-associated acute respiratory infections (ARI) and exacerbations of chronic respiratory diseases. Pandemic H1N1 influenza hit India in 2010 [3], and since then there have been regular outbreaks reported by Integrated Disease Surveillance Programs, more particularly in 2012 and 2015 when the corresponding case fatality was higher than originally reported in 2010. A recent study reported that across all age groups, a mean of 127,092 (95% CI = 64,046–190,139) annual influenza-associated respiratory and circulatory deaths may occur in India, with most deaths occurring in those aged >65 years and children <5 years of age [4].

While vaccination remains the most important intervention to prevent the development of influenza infection, the uptake of vaccines in India is low, even in priority groups at high risk for complications for influenza such as healthcare workers; pregnant females; and patients with chronic cardiac disorders, lung disorders, or diabetes [5,6,7,8,9]. Professional physician societies have only recently come up with recommendations for vaccination of patients covered in their respective domain of care [10,11,12] and the Ministry of Health, Government of India, also has only recently recommended the use of the vaccine for persons at a high risk for complications of influenza [13]. However, influenza vaccination is not included in the Universal Immunization Program (UIP) of the Ministry for a variety of possible reasons, the most important being the economic considerations for such a rollout for a country of 1.4 billion people and the health system grappling with other communicable diseases such as malaria, tuberculosis, HIV, and now COVID-19. A number of misperceptions and misconceptions exist among the common masses and even healthcare providers about influenza vaccination that adversely influence the uptake of the vaccine. In a study of healthcare workers (HCW), we found that of the recruited 1400 participants who were questioned about the knowledge, attitudes, and practices of their influenza vaccination, only 4.4% had actually received vaccination, in spite of considering influenza as a potentially severe disease with possibility of severe outcomes and despite full knowledge of a vaccine being available for its prevention [5]. Apart from concerns about the safety of the vaccines where respondents had misperceptions about the potential of the influenza vaccine to cause serious side effects such as immunological, neurological, and joint problems, many HCWs cited the ineffectiveness of the vaccine as a reason for not taking the vaccine [5]. Of the various factors that result in poor influenza vaccine uptake, we have hypothesized the poor sensitization of the healthcare providers to be the strongest impediment to the influenza vaccine uptake, with a disconnect between the perceptions and practice behaviors of the physicians [14].

While vaccine effectiveness (VE) studies have been conducted in the Western European and North American developed countries to determine the effectiveness of influenza vaccine in various seasons [15], scant data are available that address this aspect of influenza immunization in the Indian subcontinent. Even scantier are the data of the VE in patients with severe acute respiratory infection (SARI) who require hospitalization. We have preliminarily reported earlier on the VE in two influenza seasons as a part of Global Influenza Hospital Surveillance Network (GIHSN) [16,17], documenting a variable VE against influenza related SARI. We herewith report poor vaccine effectiveness (VE) as a result of genomic mismatch between the circulating strain of influenza B and the strain contained in the WHO-recommended influenza vaccine.

## 2. Methods

### 2.1. Setting and Study Population

The study was conducted in Sheri Kashmir Institute of Medical Sciences, an 850-bedded tertiary care cum referral center in the summer capital of the northern Indian State of Jammu and Kashmir. Kashmir province is located north of the 30-degree North latitude and has a unique temperate geography as against the predominantly tropical/subtropical climate in the rest of the country. We have previously documented a northern hemispherical (NH) pattern of influenza circulation in the region as compared to the dominant southern hemispherical (SH) pattern of circulation in the rest of the country [18], even as the country as a whole is located in the geographical northern hemisphere.

The study was undertaken from October 2019 to March 2020 as a part of surveillance under the aegis of the GIHSN Network [19,20], which has been conducting an annual, active-surveillance, hospital-based study since 2012 to improve understanding of influenza epidemiology to better inform public health policy decisions. Recruited patients had to be residents in the hospital’s catchment area for at least 6 months, not institutionalized, and not discharged from a hospital within 30 days of the current admission. Onset of symptoms had to be within 7 days prior to admission. Acute illness in patients aged ≥5 years had to meet the European Centre for Disease Prevention and Control (ECDC) clinical case definition of influenza-like illness (ILI), including one of the following general symptoms: fever or feverishness, malaise, myalgia, or headache, as well as including one of the following respiratory complaints: shortness of breath, sore throat, or cough [21]. The study participants involved patients (*n* = 1219) who had been admitted to the hospital from October 2019 to March 2020 and had to have been admitted within last 48 h, possibly for ARI pertaining to influenza.

For each patient, demographic and other details were collected by personal face-to-face interview and clinical case record study, which were recorded in a predefined standardized questionnaire. Collected information included age, gender, comorbidities, history of previous admissions to hospital in the last 12 months, number of visits to a general practitioner in the last 3 months, smoking habits, socioeconomic class, days from onset of symptoms to swabbing, and epidemiological week at admission. The influenza vaccination status of each patient was also collected by face-to face interview, patient records, clinical records, or registries, including the name of the vaccine received and the date of vaccination. 

### 2.2. Influenza Vaccine Composition for 2019–2020 Season

Recommended reference viruses for 2019–2020 northern hemisphere vaccines were for an A/Brisbane/02/2018 (H1N1)pdm09-like virus, an A/Kansas/14/2017 (H3N2)-like virus, and a B/Colorado/06/2017-like virus (B/Victoria/2/87 lineage); quadrivalent vaccines also included a B/Phuket/3073/2013-like virus (B/Yamagata/16/88 lineage) [22]. Participants (including children aged <9 years who are recommended to have 2 vaccine doses during their first vaccination season) were considered vaccinated if they received ≥1 dose of influenza vaccine ≥14 days before illness onset.

### 2.3. Laboratory Investigations

Nasal or nasopharyngeal swabs were collected from patients <14 years of age, whereas from patients aged >14 years, throat and nasal swabs were collected in viral transport medium (Hi-media) and were processed immediately (within 3–4 h) at the Influenza laboratory of Sher-i-Kashmir Institute of Medical Sciences. Samples were analyzed for diagnostic purposes using real-time RT PCR (Applied Biosystems) employing the CDC protocol [23] for detection of influenza. Influenza A-positive samples were further subtyped using primers and probes for A/H1N1pdm09 and A/H3, and influenza B positive samples were subtyped into B/Yamagata and B/Victoria lineages. Haemagglutinin (HA) sequencing and phylogenetic analysis was carried out using standard assay procedures at the Cnr Virus Des Infections Respiratoires France Sud, Lyon, France, and the nucleotide sequences were submitted to the GenBank database under accession numbers EPI 180439, EPI 1804279, EPI 1804213, EPI 1804207, EPI 1804169, EPI 1804219, EPI 1804291, EPI 1804301, EPI 1804145, EPI 1804299, EPI 1804297, EPI 18004189, and EPI 1804155 for influenza B (Victoria lineage). Representative A/H1N1 nucleotide sequences were submitted to the Genbank database under accession numbers EPI 1804129 and EPI 180435, whereas the A/H3N2 sequences were submitted to the database under the accession numbers EPI 1803979 and EPI 1696367.

### 2.4. Estimation of Vaccine Effectiveness (VE)

VE was estimated using the test-negative design as (1 − odds ratio (OR)) × 100, where the OR (odds ratio) was calculated by multivariable logistic regression comparing the vaccine coverage rates between influenza-positive and influenza-negative cases, after adjusting for potential confounders. Estimates were adjusted for age group, sex, and chronic disease using logistic regression. Categorical variables have been summarized as percentages, and a *p*-value of <0.05 was considered significant. All analysis were performed using Stata Version 15.0.

### 2.5. Ethics Approval

The study is a part of the GIHSN project, wherein all subjects gave their informed consent for inclusion before they participated in the study. The study was conducted in accordance with the Declaration of Helsinki, and the protocol was approved by the Ethics Committee of Sheri Kashmir Institute of Medical Sciences, Srinagar vide protocol IEC/SKIMS Protocol #88/2015 vide SIMS 1131/IEC-SKIMS/2016-128, dated 9 January 2016.

## 3. Results

**Figure 1:**
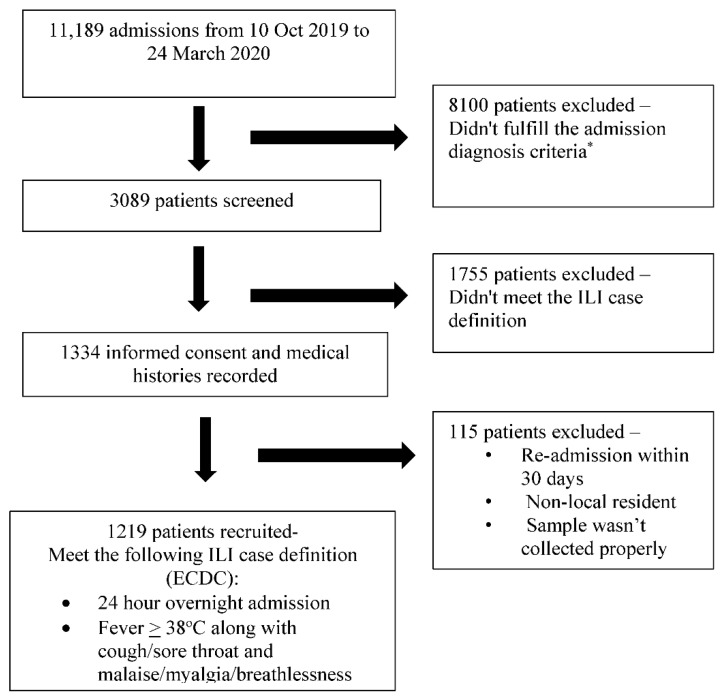
The selection of the patients for the study. The * admission diagnostic criteria as per the GIHSN format, included the following: For those aged 5 years old or more: acute upper or lower respiratory disease, acute myocardial infarction or acute coronary syndrome, acute asthma or exacerbation, acute heart failure, pneumonia and influenza, bronchitis and exacerbations of chronic pulmonary obstructive disease, acute respiratory failure, acute metabolic failure (diabetic coma, renal dysfunction, acid–base disturbances, alterations to the water balance), altered consciousness, convulsions, febrile convulsions, syncope and collapse, dyspnea, respiratory abnormality, shortness of breath, respiratory abnormality (not otherwise specified), respiratory symptoms/chest symptoms, fever or fever of unknown origin or non-specified cough, sepsis, systemic inflammatory response syndrome. For less than 5 years: acute upper or lower respiratory disease, dyspnea, breathing anomaly, shortness of breath, tachypnoea (polypnea), acute asthma or exacerbation, pneumonia and influenza, acute respiratory failure, acute heart failure, altered consciousness, convulsions, febrile convulsions, fever or fever of unknown origin or non-specified, cough, gastrointestinal manifestations, sepsis, systemic inflammatory response syndrome (not otherwise specified), nausea and vomiting.

Between October 2019 and March 2020, 3089 eligible admissions were identified of the total admissions of 11,189. Of these, 1224 met the selection criteria, and 1219 cases were eventually included in the study, with four of the excluded patients not belonging to the catchment area (non-residents) and one patient being institutionalized (Figure 1). The included 1219 patients (males *n* = 571, age range 5–107 years; median = 50 years) presented with respiratory symptoms of varying duration (1–5 days, median 3 days), and the illness was severe enough to warrant hospitalization in the opinion of the treating clinicians. The various symptoms experienced by the patients are depicted in Table 1, and all satisfied the ECDC case definition for ILI. Nearly all patients were adults, with the majority (*n* = 801, 65%) aged 18–64 years and 360 (30%) participants aged >65 years. About 71% (*n* = 866) of the participants had some comorbidity, 37.6% having more than one comorbidity. The comorbidities included cardiovascular disease/hypertension (*n* = 533; 43.7%), underlying cancer (*n* = 132; 1.8%), diabetes (*n* = 216; 17.7%), COPD (*n* = 186; 15.2%), chronic kidney disease (*n* = 132; 10.8%), rheumatological disease (*n* = 38; 31.0%), neuromuscular disease (*n* = 31; 2.5%), cirrhosis (*n* = 18; 1.5%), and bronchial asthma (*n* = 15; 1.2%). Pregnant females constituted 4% (*n* = 48) of the cases. Influenza circulation peaked during the Jan–Feb months of 2020, with 67% of the cases detected during these 8 weeks.

**Figure 2:**
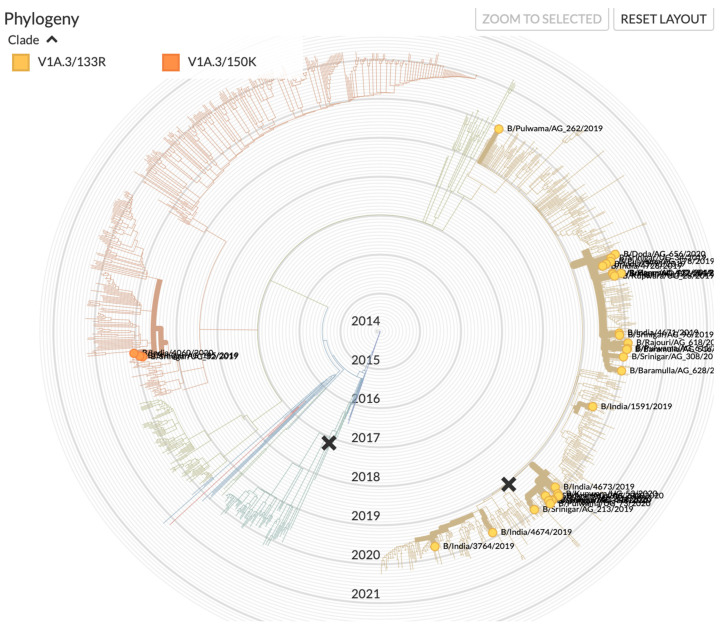
Phylogenetic tree of B/Victoria of samples in relation to the samples from India in the period under study. (source: www.gisaid.org accessed on 18 June 2021).

Influenza virus was detected in 338 (27.7%) patients. Influenza B was the most commonly identified virus, accounting for 49.7% (*n* = 167) of total influenza positives, followed by influenza A/H1N1 (47.6%; *n* = 160) and influenza A/H3N2 (4.4%; *n* = 15). Mixed infection was identified in four patients (A/H1N1 + influenza B). Upon subtyping, all the influenza B samples were found to be of the B/Victoria lineage. Sequencing of the HA gene revealed that the strain belonged to subclade V1A.3/133R with deletion of residues 163–165 and substitutions E128K, G133R, and K136E in HA1 (Figure 2). Two of the isolates (EPI 1,804,293 and EPI 1,804,351) belonged to the subclade V1A.3/150K. The influenza A/H1N1 subtype belonged to the clade 6b.1/183P-5a, whereas the A/H3 subtype belonged to the clade A1b/94N.

Around 8% (*n* = 99) of the patients had received the seasonal influenza vaccine for the 2019–2020 season; the majority (65%) of these were aged 50 years and above. The proportion of vaccinated participants was 6.8% among influenza-positive cases compared with 8.6% among influenza-negative participants (Table 2). Of the 99 vaccinated patients, 23 (23.2%) developed influenza infection compared to 313 (28%) among the unvaccinated (*n* = 1120; *p* = 0.314). Unadjusted VE for any influenza strain among age group 18–49 years was 23% (95% CI −68 to 65). After adjusting for age group, underlying comorbidity, sex, and number of days from illness onset to enrollment, VE against any influenza strain for all ages was 13% (95% CI −42 to 47), and VE against influenza A/H1N1 virus was 55% (95% CI −66 to 81), whereas it was poor against influenza B (Table 2). None of the patients who had an adverse consequence of their illness had received vaccination prior to the development of the illness, despite having comorbidities that would have put them at a high risk for complications for influenza-related complications.

## 4. Discussion

Our data revealed a poor overall VE of the vaccination against any influenza, being particularly low against influenza B viral infection, which was the dominant circulating strain, with a moderate effectiveness against the A/H1N1pdm09 strain. These results complement our earlier preliminary studies of influenza vaccine effectiveness conducted as a part of the Global Influenza Hospitalized Network (GIHSN) network in 2015–2016, wherein we demonstrated an overall adjusted VE against influenza-related hospitalization of 16.3% (95% CI, 0.4 to 29.7); adjusted VE against hospital admission with influenza was 16.2% (95% CI, −3.6 to 32.2) overall, 23.0% (95% CI, −3.3 to 42.6) against influenza A/H1N1/pdm09, and −25.6% (95% CI, −86.3 to 15.4) against influenza B/Victoria lineage [5]. Overall, VE in 2016–2017 was 27.24 (95% CI 15.62–37.27) [6]. While VE can vary depending on a number of factors, recent studies show that flu vaccination reduces the overall risk of the flu illness by between 40% and 60% among the overall population during seasons when most circulating flu viruses are well-matched to the flu vaccine [15,24,25,26].

Our results for the period under study are at variance with other studies. For example, interim VE in 2019/20 in six European studies from September 2019 to January 2020 against influenza B infection was >60% among all ages in primary care [27], with a lower VE among those aged 18 to 64 years. However, sample size was lower in this age group, and the low VE may possibly be a result of random variation. The high overall interim VE against influenza B was comparable with 2019/20 in Canada (69%) [28], whereas overall VE in the US against B/Victoria was slightly lower at 50% [29]. The comparative VE in the European Union, the United States, and Canada is depicted in Table 3.

Vaccine effectiveness against influenza has been variably reported from various studies and is influenced by a number of pathogen and host factors. In a meta-analysis that assessed VE according to various subtypes of the influenza virus, pooled VE against influenza B was 54%, with similar estimates across most of the studies [30]. Prior to 2015, VE estimates specific to the two lineages of influenza B were infrequently reported, and as such, it was not possible to assess lineage-specific protection. However, recent analysis of lineage-specific data of eight seasons from Canada’s Sentinel Practitioner Surveillance Network found VE against influenza type B exceeded 50%, regardless of vaccine lineage match with circulating viruses [31]. This cross-lineage protection has been observed in other studies as well, but it is not consistent across all seasons, and the relative magnitude of the protection across the two lineages of influenza B varied [32,33].

Although we have previously documented co-circulation of the Victoria and Yamagata lineages of influenza B in our area [34], only B/Victoria was detected in the cases in the current study. Upon genomic study, we detected B/Victoria-lineage viruses with a deletion of two or three amino acids at positions 162 and 163 or 162–164 in HA, respectively, which are antigenically distinct from the vaccine strain recommended by the WHO for the 2019/20 NH season. HA sequencing of our samples revealed that most HA genes belonged to subclade V1A.3/133R with deletion of residues 163–165 and a K136 substitution in HA1. Two of the isolates belonged to the subclade V1A.3/150K. Antigenic drift is a well-documented factor that reduces vaccine protection and contributes to VE variation across and within virus subtypes and lineages. This is a particular concern for influenza A/H3N2 viruses, which have a higher rate of antigenic evolution compared with influenza A/H1N1 or influenza B [35]. When circulating viruses do not match the vaccine strain, vaccination provides little to no protection [36,37,38]. Optimal vaccine strain selection is challenging because the decision must be made 6–8 months in advance of vaccine distribution. Recently developed phylodynamic prediction models using global surveillance data have the potential to improve strain selection (and subsequently VE) by identifying virus clades likely to predominate in the future [39].

Globally, most of B/Victoria-lineage influenza viruses detected during the eight influenza seasons that followed the 2009–2010 season were antigenically closely related to the vaccine strain B/Brisbane/60/2008 [40]. The influenza vaccine strain selected for the Victoria lineage in 2019–2020 was the B/Colorado/06/2017 strain, which was nested within clade 1A.1 with a 2-aa deletion. Antigenic characterization of Victoria viruses has shown that some 3-aa deletion strains reacted poorly with antisera raised against the Victoria clade 1A.1 vaccine strain (with a 2-aa deletion), but generally reacted better to sera raised against viruses with no deletion, suggesting that Victoria viruses are undergoing antigenic diversification [22,40]. The presence of 3-aa deletions in our strains could have reacted poorly with the ferret antisera against the clade 1A.1 contained in the vaccine. This divergence of the circulating strains from the vaccine strain likely contributed to the poor vaccine effectiveness of the vaccine against influenza B in the 2019–2020 season. Such phenomena would have serious consequences for the composition of the vaccine in the forthcoming seasons and in periods of co-circulation of the different strains. The circulation of three or more distinct influenza B viruses would drastically complicate influenza vaccine formulation and has huge implications for the strain selection for vaccine.

Biannual reports on VE in influenza are provided by Global Influenza VE (GIVE) Collaboration, and these are contributory to the selection of the vaccine strains for the forthcoming NH and SH seasons. The subsequent 2020 vaccine composition recommendation for the Southern Hemisphere reflected the change in the circulating strain, and thus a 3-aa deletion strain in clade 1A.2 (B/Washington/02/2019-like) was selected to replace the previous 2-aa clade 1.A1 (B/Colorado/06/2017-like) vaccine strain [41]. It is possible that with the continued cocirculation of these deletion variants, a reduced cross reactivity could potentially result in the divergence of B/Victoria lineages into two or more antigenically distinct variants [40]. Subsequently compared with the NH 2019-2020 trivalent vaccine recommendations, all components for the 2020-2021 trivalent vaccine were changed. For influenza A/H1N1/pdm09, WHO recommended A/Guangdong-Maonan/SWL1536/2019(H1N1)pdm09-like virus for egg-based vaccines and A/Hawaii/70/2019 (H1N1)pdm09-like virus for cell-based or recombinant-based vaccines, which are 6B.1A5A viruses, harboring additional D187A and Q189E substitutions. For influenza A(H3N2), the WHO recommended A/HongKong/2671/2019 (H3N2)-like virus for egg-based vaccines and A/HongKong/45/2019 (H3N2)-like virus for cell-based or recombinant-based vaccines, both 3C.2a1b + T135K-B viruses, harboring S137F, A138S, and F193SHA substitutions. For the 2020 Southern Hemisphere influenza vaccine, the WHO recommended for both 2020-2021 NH trivalent and quadrivalent vaccines a B/Washington/02/2019-like (B/Victoria lineage) virus (a three amino acid deletion virus), and additionally, a B/Phuket/3073/2013-like (B/Yamagata lineage) virus for the quadrivalent vaccine [41,42]. The incorporation of Washington/02/2019-like (B/Victoria lineage) is in consonance with our findings of a vaccine mismatch in the previous flu season. Combining the CDC Vic deletion assay with the CDC Flu rRT-PCR Dx Panel Influenza B Lineage Genotyping Kit will allow for rapid identification of the distinct influenza B viral genetic groups B/YAM, B/VIC V1A, V1A-2DEL, and V1A-3DEL [43].

Currently, influenza vaccines are supposed to confer protection on the basis of antibody responses to HA and NA proteins of the virus, which are rapidly evolving, and hence the vaccine needs to be strain matched with the circulating strain. However, predictions of strains that will circulate are imperfect, and manufacturing of vaccines with the predicted circulating strain takes months. Universal influenza vaccines target highly conserved antigens of the influenza virus and have been shown to be effective over short periods of time following vaccination in proof-of-concept studies. Recent data have emerged that have demonstrated that a broad, powerful, and long-lasting immune response for a universal vaccine for both influenza A as well as influenza B viruses [44]. The immune protection in the form of antibody and T-cell responses lasted about a year without boosting. Immunization for influenza B involved influenza B nucleoprotein (B/NP)-rAd conferred and protection against challenge with influenza B viruses of mismatched HA lineages [44]. Similarly, there is an interest in development of alternate platforms of vaccine manufacturing so that the turnaround time of manufacturing is faster, which would result in a better match between the circulating and the vaccine strain. In this connection, alternate strategies include using cell-based vaccines, adjuvanted vaccines, recombinant vaccines, high dose vaccines, etc., in order to achieve a closer match between the circulating and the vaccine strain for a better VE and a better immunological response to the administration of the vaccine [45]. mRNA-based influenza vaccine trials are underway currently and have been initiated recently [46]. Influenza vaccine strains traditionally have been grown in embryonated chicken eggs since the 1940s, and most inactivated influenza vaccines are still manufactured in eggs. In recent years, evidence has emerged that egg passage generates strong selection pressure for antigenically important mutations in influenza A(H3N2) viruses [47]. A link between egg adaptation and reduced VE is provided by an ecologic analysis showing a significant negative correlation between vaccine virus egg adaptation and VE against influenza A(H3N2) from 2010–2015 [47].

None of our patients had a clear history of previous vaccination for influenza. The effect of prior vaccination history is most pronounced (and variable) for VE against influenza A(H3N2). In general, VE against influenza A(H3N2) viruses is highest among those vaccinated in the current season only and lower among those vaccinated in both the current and prior seasons, although this pattern is not consistent [48,49,50]. For A(H1N1)pdm09 and type B influenza infections, VE is generally similar, regardless of prior season vaccination status. In some seasons, residual protection from prior season vaccination has been observed for type B infections.

Apart from the matching degree of circulating strains and vaccine strains, VE is also affected by the immunogenicity of vaccine. Although a well-developed global influenza surveillance network has been established to try closely match the circulating and the vaccine strain, the prediction of annual vaccine strains continues to remain a huge challenge. Thus, it would be important to improve the immunogenicity of vaccines in case there is a mismatch between the vaccine strains and the circulating strains. Factors that may affect the immune response to influenza vaccination including age, gender, use of adjuvants, and the delivery mode. In addition, individuals sharing the same features might respond differently to the same vaccine, indicating a role of genetic factors in the immune response to the vaccine. A relationship between single nucleotide polymorphism (SNP) in human leukocyte antigen (HLA) and the immune responses to influenza vaccination has been demonstrated [51]. More recently, when genome-wide association studies (GWAS) were employed, two novel candidate missense variants, *ZBTB46* rs2281929 and *IQGAP2* rs2455230, were found to be associated with the immune response to influenza vaccination among the Chinese population [52]. This opens a new arena for influenza vaccine effectiveness and points to the possibility of a host-determined response to the administration of the influenza vaccine. Identifying these variants will provide more evidence for future research and improve the individualized influenza vaccination program.

## 5. Limitations of the Study

The present study is a single center study that would argue against the generalization of the results for the country or the region. However, the fact that influenza B circulation is evolving in the manner described places emphasis on further studies for requirement of possible expansion of the influenza B strains in future vaccines. Further, the study was primarily focused on in-patients suffering from a potential source of bias that could affect VE estimates from inpatient test-negative design studies. Patients with certain chronic conditions, such as chronic pulmonary, renal, cardiac, or metabolic disorders, may also require admission for non-ARI events, e.g., decompensation of their underlying respiratory or cardiac condition, that may not necessarily be associated with influenza infection. If these events meet the study inclusion criteria, these subjects would be enrolled as controls and in fact serves as indirect evidence for the over-representation of patients with chronic comorbidities among controls in inpatient studies employing test-negative design [53]. Such a selection bias is possible and cannot be entirely eliminated. Moreover, it is assumed that patients with such comorbidities would have a high vaccination rate that would result in bias. Although no national data are available on vaccine coverage against influenza as the vaccination in not included in the Universal Immunization Program of the Ministry of Health and Family Welfare, we have earlier documented an overall poor uptake of influenza vaccine, in studies with smaller numbers, in patients/people who are at high risk for influenza complication due to underlying comorbidity or condition [5,6,7,8,9]. An overall low coverage would minimize over-representation of the vaccinated individuals among the controls. Regardless of the vaccine effectiveness, our study strives to bring forth the possibility of a divergence of influenza B strains that might eventually play a part in future vaccine strain selection.

## 6. Conclusions

In conclusion, our data emphasize continued genomic surveillance for influenza for appropriate matching of the vaccine and the circulating strain of influenza B, as well as exploration of newer platforms for development of uniformly effective vaccines that are not only effective against no-matched strains but also confer a longer lasting protection against influenza.

## Figures and Tables

**Table 1:** Clinical symptoms of participants at presentation.

Symptoms	Vaccinated (*n* = 99)	Non-Vaccinated (*n* = 1120)
Fever	57 (57.5)	781 (69.7)
Malaise	65 (65.6)	617 (55)
Headache	47 (47.5)	543 (48.4)
Myalgias	68 (68.6)	825 (73.6)
Cough	92 (92.9)	1039 (92.7)
Sore throat	54 (54.5)	505 (45)
Breathlessness	74 (74.7)	789 (70.4)

**Table 2:** Vaccine effectiveness against the trivalent influenza vaccine for the season 2019–2020 among different age groups.

Age Group (years)	Influenza Positives (N)	Influenza Positives Vaccinated (N)	Influenza Negatives Total (N)	Influenza Negatives Vaccinated (N)	Unadjusted VE (%)	95% CI		Adjusted VE (%)	95% CI	
All ages	336	23	883	76	22	−27 to 52	0.315	14 *^,†^	−41 to 47	0.549
5–17	28	0	35	0	-	-		-	-	
18–49	162	9	351	25	23	−68 to 65	0.508	24 ^‡^	−66 to 66	0.485
50–64	65	3	219	19	49	−78 to 85	0.290	49 ^‡^	−78 to 85	0.291
>65	81	11	278	32	−21	−152 to 42	0.614	−67 ^‡^	−145 to 44	0.683
Virus type										
A/H1N1	155	6	883	76	57	0 to 82	0.050	55 *	−6 to 81	0.068
B/Victoria	163	14	883	76	0	−81 to 45	0.994	−12 *	−106 to 39	0.708

* Adjusted for age group, gender, and presence of chronic condition; ^†^ McFadden pseudo R^2^ = 0.0156, LR chi-squared (6) = 22.38 (*p* = 0.0010), Hosmer–Lemeshow test chi-squared (8) = 3.30 (*p* = 0.9144); ^‡^ Adjusted for gender and presence of chronic condition.

**Table 3:** Depiction of the comparative vaccine effectiveness against any influenza (A + B) and influenza B in the 2019-2020 season reported from other geographic locations.

Site	Influenza-Positive		Influenza-Negative		Adjusted VE, % (95% CI)
	Total	Vaccinated	Total	Vaccinated	
	N	N (%)	N	N(%)	
European Union [27]					
EU (PC) Influenza B	12	4 (NC)	313	199 (64)	60 (−69 to 90)
Influenza B/Victoria	209	5 (2)	1190	141 (12)	−12 to 86
EU (H) Influenza A	122	50 (41)	473	312 (66)	62 (41 to 76)
United States [29]					
All influenza types	1060	390 (37)	3052	1682 (55)	45 (36–53)
Influenza B/Victoria	634	211 (33)	2968	1641 (55)	50 (39–59)
Influenza A(H1N1)pdm09	336	138 (42)	3052	1682 (55)	37 (19 to 52)
Canada [28]					
All influenza types	1411	191 (14)	1397	399 (29)	58 (47 to 66)
Influenza A	731	131 (18)	1397	399 (29)	49 (34 to 60)
Influenza B	683	60 (9)	1397	399 (29)	69 (57 to 77)
Present study					
Any influenza (A + B)	336	23 (7)	883	76 (9)	14 (−41 to 47)
Influenza B/Victoria	163	14 (9)	883	76 (9)	−12 (−106 to 39)

EU = European Union; PC = primary care setting; H = hospital setting.

## Data Availability

Data are available on request from the Influenza Lab of Sheri Kashmir Institute of Medical Sciences, Srinagar, India and the corresponding author at parvaizk@gmail.com.
